# The *penetrance* R package for estimation of age specific risk in family-based studies

**DOI:** 10.1093/bioadv/vbaf154

**Published:** 2025-07-08

**Authors:** Nicolas Kubista, Danielle Braun, Giovanni Parmigiani

**Affiliations:** Department of Biostatistics Harvard T.H. Chan School of Public Health, Boston, MA 02115, United States; Department of Data Science, Dana-Farber Cancer Institute, Boston, MA 02215, United States; Department of Biostatistics Harvard T.H. Chan School of Public Health, Boston, MA 02115, United States; Department of Data Science, Dana-Farber Cancer Institute, Boston, MA 02215, United States; Department of Biostatistics Harvard T.H. Chan School of Public Health, Boston, MA 02115, United States; Department of Data Science, Dana-Farber Cancer Institute, Boston, MA 02215, United States

## Abstract

**Motivation:**

Reliable tools and software for penetrance (age-specific risk among those who carry a genetic variant) estimation are critical to improving clinical decision making and risk assessment for hereditary syndromes. However, there is a lack of easily usable software for penetrance estimation in family-based studies that implements a Bayesian estimation approach.

**Results:**

We introduce *penetrance*, an open-source R package available on CRAN, to estimate age-specific penetrance using family-history pedigree data. The package uses a Bayesian estimation approach, allowing for the incorporation of prior knowledge through the specification of priors for the parameters of the carrier distribution. It also includes options to impute missing ages during the estimation process, addressing incomplete age information which is not uncommon in pedigree datasets. Our open-source software provides a flexible and user-friendly tool for researchers to estimate penetrance in complex family-based studies, facilitating improved genetic risk assessment in hereditary syndromes.

**Availability and implementation:**

The *penetrance* package is freely available on CRAN. Source code and documentation are available at https://github.com/nicokubi/penetrance.

## 1 Introduction

Precision prevention and early detection of heritable diseases rely on identifying individuals at increased risk. Comprehensive germline DNA analysis panels can reliably detect pathogenic germline variants (PGVs) that may increase disease risk. In this context, an accurate estimate of age-specific risk of disease is a crucial input for counseling individuals.

A penetrance function quantifies the proportion of carriers who develop a condition by a specific age. Penetrance estimation has been extensively studied in the past for a variety of study designs. Family-based studies usually center around a proband who is typically the initial point of contact with the healthcare provider and is the individual reporting their family history. The proband is often the only genotyped individual in the family and may or may not report additional genotyping information for known relatives. Different methods for penetrance estimation have also been previously developed for family-based studies (Shin *et al.* 2020). Classical segregation analysis, for example, has been widely used to infer inheritance patterns and estimate penetrance, but it often requires large, well-characterized pedigrees and can yield imprecise estimates when sample sizes are small (Elston and Stewart 1971).

However, in our assessment, there is a lack of easily usable software for penetrance estimation for family-based studies that implements a Bayesian estimation approach. For example, the widely used software package MENDEL by Lange *et al.* (2008) offers an option for penetrance estimation which can account for a full pedigree, but is difficult to use and interpret. MENDEL requires users to become proficient with its specialized input file formats and complex syntax, which can present a learning curve for researchers primarily working within R-based analytical workflows. Other penetrance estimation software and webtools are outdated and no longer maintained. To our knowledge no other software packages for penetrance estimation are readily available today. Additionally, when analyzing a study of PGV carriers, it can be important to integrate prior knowledge from published studies, which provide important information. Currently available software packages do not provide options to easily include prior knowledge.

We introduce *penetrance*, an open-source R software package, to carry out age-specific and sex-specific penetrance estimation for complex family-based studies where carrier status is not observed for all family members. The implementation is based on Bayesian estimation methods, which allows for the incorporation of prior knowledge from existing studies and provides a flexible and easy-to-use method for penetrance estimation. For example, consider incorporating detailed age-specific penetrance data from published studies. If a large cohort study reports that carriers develop disease with approximately 10% cumulative risk by age 45 (first quartile), 25% by age 55 (median), and follows patients until age 80, with detailed information on the number of individuals at risk at each timepoint, this granular quartile information can be incorporated using the distribution_data_default parameter (see Supplementary Materials 8, available as supplementary data at *Bioinformatics Advances* online).

Our software provides a flexible and intuitive tool for researchers to estimate penetrance in complex family-based studies, facilitating improved genetic risk assessment in hereditary syndromes. While applicable across various heritable conditions, our primary motivation stems from cancer genetics, where early detection and prevention strategies significantly impact patient outcomes (Rebbeck *et al.* 2018).

## 2 Methods

### 2.1 Model

Our package implements a pedigree-based Bayesian framework for estimating age-specific penetrance in family-based studies. This approach focuses on modeling disease risk for a single disease at a time, though our methods could be extended to multiple diseases in future work.

The statistical model integrates critical population-level parameters, including carrier prevalence in the general population and baseline age-specific disease probabilities for non-carriers.

For penetrance modeling, we use a modified Weibull distribution with parameters θ=(α,β,γ,δ), where scale (α>0) and shape (β>0) are standard Weibull parameters, threshold (δ>0) accounts for minimum onset age, and asymptote (0<γ<1) represents lifetime disease probability. This parameterization creates a flexible cumulative distribution function representing disease onset probability over time. We use a parametric Bayesian approach to estimate the posterior distribution of θ. Since direct calculation is intractable due to the complexity of the likelihood function and unobserved variables, we use a fine-tuned adaptive Markov Chain Monte Carlo (MCMC) method (Metropolis *et al.* 1953) to approximate the posterior distribution.

### 2.2 Package usage

**Figure 1.**
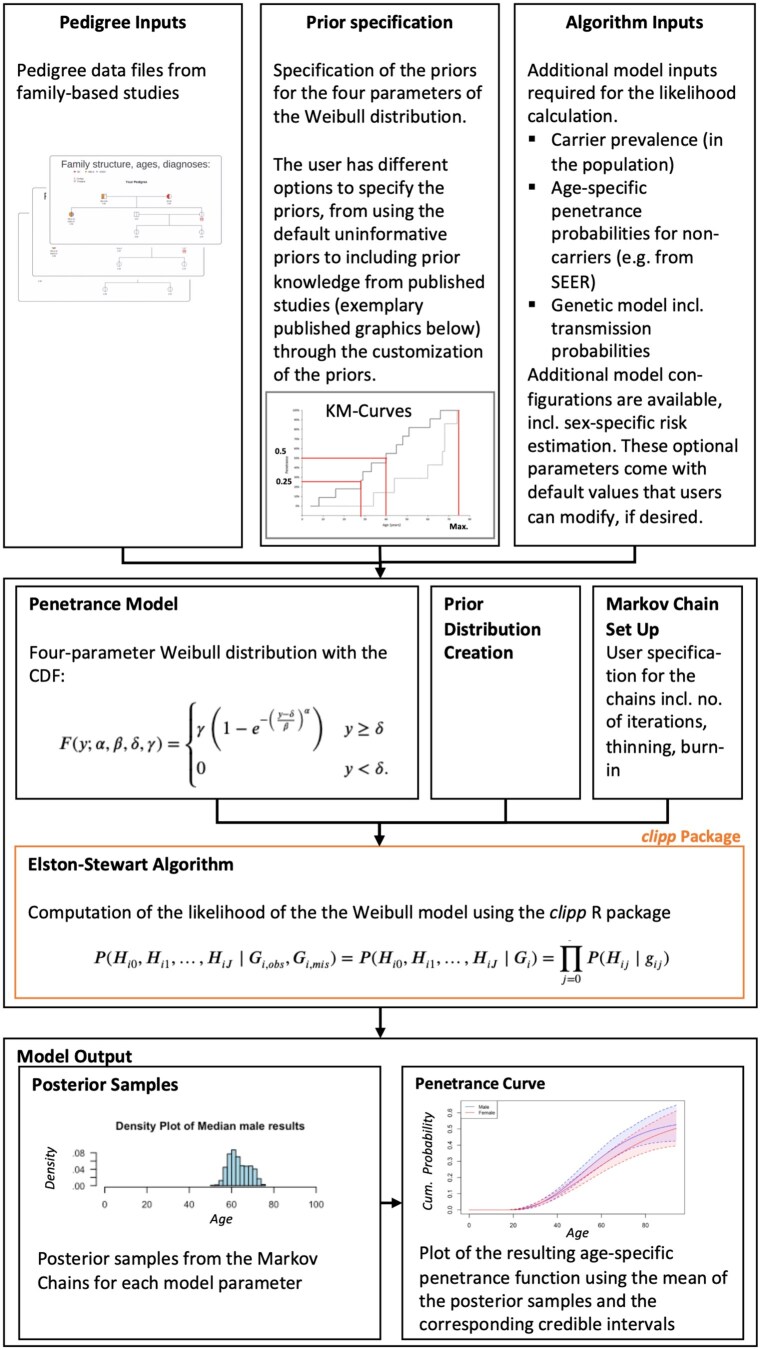
Workflow of the *penetrance* Package with dependency on *clipp*.

The methods discussed above are implemented in an easy-to-use R software package, available on CRAN. The package workflow includes three main parts: (i) the user input, including family data in the form of pedigrees, and the specifications of the options for the penetrance estimation, (ii) the estimation of the posterior distribution using the MCMC approach, and (iii) the output of the results in the form of the samples from the approximated posterior distribution, i.e. the estimated penetrance function. Figure 1 shows the package workflow.

The main input is the family pedigree data reported by the proband. The pedigree file is an R data frame object. Each family tree is represented by a single data frame that includes the following columns: *PedigreeID, ID, Sex, MotherID, FatherID, isProband, CurAge, isAff, Age*, and *Geno*. Each row represents a unique family member, with family relationships established through parent ID references. The *isProband* column (value 1) designates the individual reporting family history. Disease status is indicated through *isAff* (1 = diagnosed, 0 = unaffected, NA=unknown) and *Age* (age at diagnosis or NA). Carrier status is recorded in *Geno* (1 = carrier, 0 = non-carrier, NA=unknown). The column *CurAge* indicates the censoring age, which is the current age if the person is alive or the age at death if the person is deceased. Missing ages are allowed and can be coded as NA or left empty. More details on the structure of the pedigree required for *penetrance* is available in the *Fam3Pro* (formerly PanelPRO) documentation where a similar structure is used (Lee *et al.* 2021).

To run the MCMC algorithm, the prior distributions for θ=(α,β,γ,δ) must be specified. The package provides three flexible approaches to prior specification, balancing customization with ease of use. First, the package implements reasonable default uninformative priors [First Quartile: Scaled Beta(6,3), Median: Scaled Beta(2,2), Asymptote: Scaled Beta(1,1), Threshold: Uniform(5,30)], suitable for exploratory analysis when limited prior knowledge on the shape of the penetrance curves is available. Second, users can manually adjust prior parameters by modifying the *prior_params_default* object, which is appropriate when researchers have specific beliefs about parameter distributions that do not align with default settings. Third, the package facilitates incorporation of published penetrance estimates through several supported formats: relative risk estimates (e.g. odds ratios) that inform the asymptote parameter, detailed age-specific risk data such as information extracted from Kaplan-Meier curves, or high-level age distribution data combined with overall study size. We recommend using relative risk estimates for quick incorporation of meta-analytic findings, detailed age-specific data when Kaplan-Meier curves are available from studies, and high-level data when only summary statistics are reported in those studies. This literature-based approach is particularly valuable when analyzing rare variants, as it leverages existing knowledge to improve estimation precision. Supplementary Materials 8, available as supplementary data at *Bioinformatics Advances* online provides comprehensive details on the prior elicitation process and approach used to translate published data into prior distributions.

The estimation procedure follows the approach outlined in the methods section. In addition to the pedigree data (*data*) and the specified priors (prior_params) the user inputs a carrier prevalence (*prev*) and a baseline age-specific probability of developing the disease (baseline_data). Assuming carriers are rare, the population age-specific probability of developing the disease is a good approximation for the noncarrier penetrance. Such population-level risk data usually comes from a population registry such as the Surveillance, Epidemiology, and End Results (SEER) Program for cancer in the United States (Howlader *et al.* 2020). Per default, the estimation is sex-specific, estimating two distinct set of parameters for females and males. However, the user has the option to run the estimation without considering sex, by setting *sex_specific = FALSE*. The user can provide additional inputs to the function at run-time. An overview of the options the user can specify in the main function call can be found in the Table A1, available as supplementary data at *Bioinformatics Advances* online.

The estimation requires preparation of the pedigree data, particularly handling missing age information and adjusting the data format. A penetrance function quantifies the proportion of carriers who develop a condition by a specific age. Details on how the package handles missing ages are provided in Supplementary Materials, available as supplementary data at *Bioinformatics Advances* online.

For the estimation procedure, the initial set of parameters, denoted $\theta_{0}$, is drawn from the empirical distribution of the provided data, stratified by sex and disease affection status. In each iteration of the MCMC algorithm, a new proposal is generated for the parameter vector. After generating the proposal, it is essential to ensure that each parameter falls within its respective bounds. These bounds are set to maintain realistic and meaningful parameter values based on biological plausibility (see Supplementary Materials 9, available as supplementary data at *Bioinformatics Advances* online). If any parameter falls outside these bounds, the proposal is automatically rejected, ensuring that the algorithm only considers valid parameter values for further evaluation.

Based on the proposed parameters of the Weibull distribution, *penet.fn* calculates the age-specific penetrance for every individual at each iteration of the algorithm. The *clipp* package is used to efficiently compute the likelihoods using the Elston-Stewart algorithm (Golicher 2023). It assumes a genetic model with a single biallelic locus. Age-specific penetrance probabilities are adjusted based on carrier status and affection status of the individual.

After running for the specified number of iterations, the *penetrance* function returns a comprehensive set of outputs from the MCMC estimation process. Each chain generates its own posterior samples, and these samples collectively represent draws from the target posterior distribution.

## 3 Illustrative example

To illustrate our method, we conducted a simulation study designed to mirror real-world familial colorectal cancer (CRC) patterns associated with Lynch syndrome. The simulation parameters were specifically chosen to reflect characteristics of PGVs in the MLH1 gene, one of the most common mismatch repair genes associated with Lynch Syndrome. We based our carrier prevalence on population-level MLH1 data, while the penetrance parameters (for simulating disease status) were derived from established CRC risk estimates in MLH1 carriers (Felton *et al.* 2007, Wang *et al.* 2020).

We simulated 130 probands with PGVs in the MLH1 gene, along with a total of 4604 individuals including probands (average family size= 35.4) with 428 CRC diagnoses (203 females and 225 males). The dataset, code, and resulting output of this illustration can be found in the vignette of the R package.

We generate a chain of 20 000 posterior samples and discard the first 2000 samples as burn-in (burn_in=0.1) in each plot. We do not perform any age imputation (age_imputation=FALSE) and do not attempt any ascertainment correction by removing the proband (remove_proband=FALSE).

The *penetrance* function returns the outputs discussed in the methods section and an R object that includes the posterior samples which can be used for subsequent analysis.

## 4 Conclusion

The *penetrance* open-source R package is a specialized tool for estimating age-specific disease risk in carriers of pathogenic genetic variants using family-based data. It addresses the critical need for reliable penetrance estimates in genetic counseling and risk assessment for hereditary conditions. Using a Bayesian framework with a modified Weibull distribution, the package allows researchers to incorporate prior knowledge from published studies while providing sensible defaults for those without prior information.

Key features of the package include flexible prior specification options, sex-specific penetrance estimation, and automated handling of missing age data through an optional imputation algorithm. The software implements the Elston-Stewart peeling algorithm through the clipp package for efficient likelihood calculation in complex pedigree structures. Comprehensive visualization options help researchers interpret results easily.

Computationally, the algorithm can handle pedigrees of arbitrary size, with larger pedigrees and those with many first-degree relatives of carriers generally being more informative. The statistical reliability of penetrance estimates depends on family size and structure. For smaller families, credible intervals tend to be wider and may require additional MCMC iterations. If probands are removed (remove_proband=TRUE), singleton families are automatically excluded as they do not provide age-specific risk information. When working with smaller families, users should exercise caution when interpreting results and consider using informative priors based on published literature to stabilize estimation. The package also provides convergence diagnostics through trace plots and autocorrelation functions to assess sampling adequacy.

The current implementation focuses on single-disease, single-carrier-status models with sex as the primary stratification variable, and assumes a unimodal Weibull distribution for age-specific penetrance patterns. While these design choices accommodate many hereditary syndromes, future extensions could incorporate competing risks, multiple disease outcomes, and more flexible penetrance distributions.

By combining statistical rigor with user-friendly implementation, *penetrance* provides genetic epidemiologists with an accessible tool for estimating disease risk from family-based studies. The package was developed in the context of cancer genetics research but is applicable across many hereditary conditions where accurate risk assessment is essential for clinical decision-making. The software is freely available on CRAN with comprehensive documentation and examples.

## Supplementary Material

vbaf154_Supplementary_Data

## Data Availability

This manuscript introduces *penetrance*, an innovative penetrance estimation tool. The package is available on CRAN and the source code can be accessed at https://github.com/nicokubi/penetrance.
