# Supplementary material for: The penetrance R package for estimation of age specific risk in family-based studies
Source: Bioinform Adv. 2025 Jul 8;5(1):vbaf154. doi: 10.1093/bioadv/vbaf154 (PMC12270257; doi:10.1093/bioadv/vbaf154)
Supplement: vbaf154_Supplementary_Data [file vbaf154_supplementary_data.pdf]

Supplementary Material for: Development of the *penetrance* R package for Penetrance Estimation in Family-based Studies

by N. Kubista, D. Braun, G. Parmigiani

Penetrance Package Options

Table A1. List of model options for *penetrance* (excluding prior and output options).

Argument Name	Value	Default Value	Definition
data	NA	NA	Family pedigree data frame in the appropriate format.
twins	List	NA	Identical twins or triplets in the family can be specified.
n_chains	Integer > 0	1	Number of chains for parallel computation.
n_iter_per_chain	Integer > 0	10000	Number of iterations per chain.
ncores	Integer > 0	6	Number of cores for parallel computation.
baseline_data	Dataset	Default Object	Data for baseline risk estimates.
max_age	Integer > 0	94	Maximum age considered for analysis.
remove_proband	Boolean	FALSE	Logical indicating whether to remove probands from the analysis.
age_imputation	Boolean	FALSE	Logical indicating whether to perform age imputation.
imp_interval	Integer > 0	10	The interval at which age imputation for affected and unaffected missing ages should be performed when age_imputation = TRUE.
sex_specific	Boolean	TRUE	Logical indicating whether to perform sex-specific estimation.
median_max	Boolean	TRUE	Whether to use baseline median age or max_age as upper bound for median proposal.
BaselineNC	Boolean	TRUE	If TRUE, noncarrier penetrance is assumed to be the baseline penetrance.
var	Vector	c(0.1, 0.1, 2, 2, 5, 5, 5, 5)	Vector of initial variances for the covariance matrix of the proposal distribution.
burn_in	Fraction (0 to 1)	0	Fraction of results to discard as burn-in.
thinning_factor	Integer ≥ 1	1	Thinning factor, applied to individual Markov chains.
distribution_data	List or NA	Default Object	Data used to generate prior distributions.
prev	Float	0.0001	Prevalence (of the carrier status) in the population.

Prior Elicitation based on existing studies

The package provides the user with the option to automatically generate priors based on information gathered in existing penetrance studies. Since users may often not have enough knowledge to directly define the custom parameters for the priors, we provide three options to facilitate the incorporation of different types of data that a user might be able to recover from published studies:

1. **Relative Risk Estimates:** This option uses published measures of overall relative disease risk (e.g., odds ratios [OR] or relative risks [RR]) to inform the prior on the asymptote parameter, which represents lifetime penetrance. Specifically, if an overall disease risk measure for carriers is reported, you can enter it using the parameter $ratio$. The software then multiplies this ratio by the cumulative baseline risk to determine the mean of the asymptote parameter's prior.
2. **Granular Age-Specific Risk Data:** This approach requires detailed data on the age distribution of disease diagnoses in a study. Such information may be recovered, for example, from Kaplan-Meier (K-M) curves. In the $distribution\_data\_default$ object, users must populate two rows based on the information from the study: age and at_risk. The age row should contain four time points: the earliest age of diagnosis (row name min), the age at which 25% of cases occurred (row name $first\_quartile$), the age at which 50% of cases occurred (row name $median$), and the maximum age (row name max). The at_risk row must contain the corresponding number of carriers still under observation at each of these ages (see Figure A1 - Panel a). Based on these inputs the algorithm then computes the parameters α and β for the beta distributions of the priors for the median, first quartile, and asymptote (see Table A2).
3. **High-level Age-Specific Risk Data:** This option is suitable when only the age distribution of diagnoses and the overall study size are available (but not the number of people at risk at every time point). Users populate just the age column in the $distribution\_data\_default$ object with the same four time points described above (using identical row names) and provide the total number of carriers through the $sample\_size$ parameter. The package then calculates the number of at-risk carriers at each age point using predefined proportions: 90% of the total sample size at the first quartile age, 50% at the median age, and 10% at the maximum age see Figure (A1 - Panel b).

```r
distribution_data_a <- data.frame(
  row.names = c("min", "first_quartile", "median", "max"),
  age = c(25, 40, 60, 90),
  at_risk = c(999, 900, 500, 100)
)
```

(a) Example configuration with user-provided prior information on ages and resp. number of individuals at risk at those ages.

```r
distribution_data_b <- data.frame(
  row.names = c("min", "first_quartile", "median", "max"),
  age = c(25, 40, 60, 90),
  at_risk = c(NA, NA, NA, NA)
)

sampe_size <- 1000
```

(b) Example configuration with user-provided ages and the total sample size.

Fig. A1. Structure of the input object for prior information.

Table A2. Automatic prior elicitation using user inputs in distribution data default (Setting 3).

Model Parameter	Distribution	Parameter 1 (Alpha)	Parameter 2 (Beta)
First Quartile	Scaled Beta	From *distribution_data_default*: Normalized first quartile * number of individuals at risk at the first quartile	Number of individual at risk at the first quartile - alpha
Median	Scaled Beta	From *distribution_data_default*: Normalized median * number of individuals at risk at the median	Number of individual at risk at the median - alpha
Asymptote	Scaled Beta	From *distribution_data_default*: Normalized max. age * number of individuals at risk at the max. age	Number of individual at risk at max. age - alpha
Threshold	Uniform	0	From *distribution_data_default*: Min. age

Thresholds for the Sampling of Proposals

Parameter bounds in our MCMC algorithm ensure biological plausibility while maintaining computational efficiency. These thresholds prevent the exploration of biologically impossible parameter combinations while ensuring interpretable results. The specific bounds are detailed in Table B3.

**Parameter**	**Condition**
Asymptote	$0 \leq$ Asymptote ≤ 1
Threshold	$0 \leq$ Threshold ≤ 100
Q_{50} (Median)	$Q_{50} \geq Q_{25}$<br>$Q_{50} \leq$ Median Age of Onset of the SEER Baseline or<br>$Q_{50} \leq$ max_age
Q_{25} (First Quartile)	Threshold $\leq Q_{25} \leq Q_{50}$

Table B3. Conditions for the parameters irrespective of sex.

Age Imputation

By default, the package assumes that the age information on probands and relatives are complete and no age imputation is required. In the case where an individual lacks an age of diagnosis or censoring age, their age-related contribution is omitted from the likelihood calculation. To address datasets with missing age data, the *penetrance* package provides an option to automatically impute missing ages of diagnosis or censoring ages. The imputation is based on the individual's affection status, sex, and degree of relationship to the proband, who is typically a carrier. We address missing ages of diagnosis in affected individuals by computing posterior carrier probabilities to impute missing ages by sampling from the estimated penetrance function (for likely carriers) or the provided baseline (for likely noncarriers). For unaffected individuals with missing ages, we impute censoring ages from the empirical age distribution of unaffected individuals in the data and update these imputed ages periodically during MCMC to incorporate uncertainty into parameter estimates. The frequency with which ages are imputed affects the run time and can be specified using the option $imp\_interval$. The default is $imp\_interval = 10$, which means that age imputation is performed at every 10th iteration.

Illustrative Example Outputs

```
# Set the prevalence for the gene of interest (e.g. MLH1 here)
prevMLH1 <- 0.00045

# Run the estimation procedure with the default settings
out <- penetrance(
  pedigree = dat, n_chains = 1, n_iter_per_chain = 20000,
  baseline_data = baseline_data_default, prev = prevMLH1,
  prior_params = prior_params_default, burn_in = 0.1,
  age_imputation = FALSE, remove_proband = FALSE
)
```

Fig. B2. Exemplary code for running *penetrance* for simulated data for MLH1 and CRC.

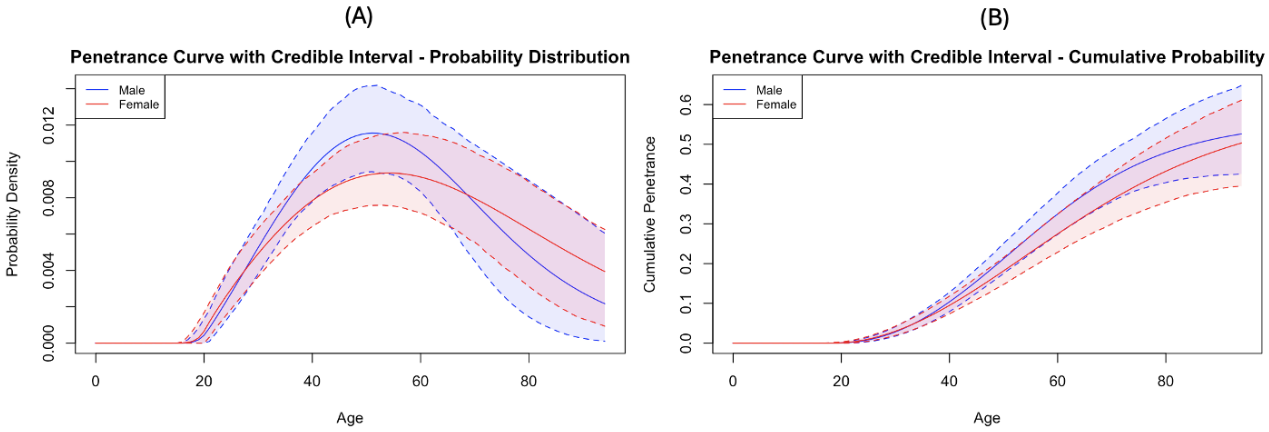

Fig. B3. Age-specific absolute risk (A) and cumulative risk (B) for CRC and MLH1 for females and males with default prior parameters for simulated data.
